# Comparison of medical student performance in summative undergraduate paediatric examinations and a clinician-designed minimum accepted competency (MAC) assessment

**DOI:** 10.1186/s12909-021-02642-7

**Published:** 2021-04-07

**Authors:** Patrick McCrossan, Dara O’Donoghue, Alf Nicholson, Naomi McCallion

**Affiliations:** 1 Queen’s University Belfast, 97 Lisburn Rd, Belfast, BT9 7BL UK; 2 School of Medicine, RCSI Bahrain, Building No. 2441, Rd 2835, Busaiteen 228, Manama, 15503 Bahrain; 3 Department of paediatrics, RCSI, 123 St StephenIs Green, Dublin 2, Ireland

**Keywords:** Medical education, Paediatrics, Assessment, Competence

## Abstract

**Background:**

It is recognised that newly qualified doctors feel unprepared in many areas of their daily practice and that there is a gap between what students learn during medical school and their clinical responsibilities early in their postgraduate career. This study aimed to assess if undergraduate students and junior paediatric doctors met a Minimum Accepted Competency (MAC) of knowledge.

**Methods:**

The knowledge of undergraduates and junior paediatric doctors was quantitatively assessed by their performance on a 30-item examination (the MAC examination). The items within this examination were designed by non-academic consultants to test ‘must-know’ knowledge for starting work in paediatrics. The performance of the students was compared with their official university examination results and with the performance of the junior doctors.

**Results:**

For the undergraduate student cohort (*n* = 366) the mean examination score achieved was 45.9%. For the junior doctor cohort (*n* = 58) the mean examination score achieved was significantly higher, 64.2% (*p* < 0.01). 68% of undergraduate students attained the pass mark for the MAC examination whilst a significantly higher proportion, 97%, passed their official university examination (*p <* 0.01). A Spearman’s rank co-efficient showed a moderate but statistically significant positive correlation between students results in their official university examinations and their score in the MAC examination.

**Conclusion:**

This work demonstrates a disparity between both student and junior doctor levels of knowledge with consultant expectations from an examination based on what front-line paediatricians determined as “must-know” standards. This study demonstrates the importance of involvement of end-users and future supervisors in undergraduate teaching.

## Background

Every year a fresh group of medical graduates start work and take on responsibility for clinical decision-making and the treatment of patients. Senior medical staff provide guidance and supervision, but newly qualified doctors are expected to assume a degree of independence by virtue of their undergraduate medical training [1]. Despite this, it is recognised that early-career junior doctors identify a number of gaps between what they were taught during their undergraduate years and their clinical work as a doctor [2]. There is also a reported discrepancy between graduates’ self-assessment and their educational supervisor’s assessment of their practice, suggesting either a lack of clarity of expected standards or elements of inter-observer variability [3]. A General Medical Council (GMC) report exploring the extent to which United Kingdom (UK) medical graduates are prepared for practice’ recognised that newly qualified doctors feel unprepared in many areas of their daily practice and recommended transition interventions, such as assistantships or work-shadowing, to address this [4].

Undergraduate curricula are generally designed to provide a broad base of paediatric knowledge for doctors in all fields, but there are potential difficulties [5]. For specialities such as Paediatrics, where there is often a 12-month interval between qualification and starting clinical practice, there is likely to be significant knowledge decay over this time [6]. Worryingly, one study has shown that a year after passing their undergraduate paediatric examination, students’ marks in the same examination decrease by 50% [7]. A year’s experience working in general clinical practice may consolidate clinical skills, but it appears that undergraduate specialty knowledge is often poorly retained. This could have implications for both undergraduate content and methods of learning but also for postgraduate training programs and induction.

Many undergraduate curricula and assessment strategies are designed by academic doctors employed by universities, and there is little evidence of input from ‘non-faculty’ clinicians [8]. However, after qualifying it is often the ‘non-faculty’ clinicians supervising them, who set the standard of what is expected in their clinical practice [9]. While clinical knowledge and skills are not the only desired outcomes of an undergraduate program, they remain core to most courses [5]. At undergraduate level, contributions from non-faculty clinicians are often informal. This contrasts with the postgraduate exam approach that actively encourages and seeks out non-faculty clinician input [10]. There is a paucity of published literature on what level of knowledge is expected of new trainees by clinical consultants working in frontline paediatrics. While undergraduate and postgraduate training curricula are explicit, there is no clear roadmap or specific clinical guidance documenting what is expected of the trainee as they start in paediatrics, other than an extrapolation from an undergraduate university assessment- which may reflect a more general graduate requirement.

This study aims to evaluate how undergraduate students perform in a knowledge-based examination set by non-faculty clinicians at a level that they deemed was “must -know”, i.e. the basic level of knowledge they would expect from a junior doctor (Senior House Officer (SHO)) starting in paediatrics.

## Methods

This was a study of performance in a novel paediatric examination by undergraduate students from two large Irish medical schools and paediatric junior doctors. Ethical approval for the project was obtained from the Royal College of Surgeons in Ireland (RCSI) Research Ethics Committee [REC 1129b], the Royal College of Physicians of Ireland (RCPI) Research and Ethics Department [RCPI RESCAF 51] and Queen’s University Belfast (QUB) Research and Ethics Committee [18.01].

### Devising a minimum accepted competency (MAC) paper

Clinicians registered with the RCPI (Paediatric division) were contacted by e-mail on 01/08/2015 with a request to provide questions for use in this examination. They were asked to generate questions based on “must know” information that, in their opinion, was necessary for every junior doctor starting their first post in paediatrics. Each clinician was asked to submit examination questions in ‘multiple choice’ (MCQ) or ‘true/false’ format. One follow-up e-mail was delivered on 01/02/2016 and no further submission were permitted beyond 01/05/2016. Submissions from clinicians who held an academic position at a university were excluded. The questions were reformatted to a ‘single best answer’ MCQ structure to match the question format in use at the time for paediatric undergraduates at RCSI.

An academic trained in assessment and question writing, who was not directly involved in the study, reviewed the questions for clarity and language, however neither content nor level of difficulty were changed. A bank of questions was created, and a random number generator was used to choose 30 questions to form the research examination (MAC) paper (01/06/2016). The examination paper was limited to 30 questions to maximise participant recruitment.

### Creating a passing score

On 17/06/2016 the questions were standard-set by the undergraduate academic paediatric faculty of the RCSI at a standard-setting meeting for the university’s paediatric written examination. Academic staff present included the professor of paediatrics, the associate professors of paediatrics and paediatric clinical lecturers. The academic team had experience of standard-setting using the Angoff technique, whereby examiners estimate what proportion of the ‘minimally competent’ or ‘borderline’ candidates they would expect to correctly answer a given question [4]. The mean of those estimates becomes the standard set for that question. The mean for all of the questions then becomes the ‘passing’ or ‘cut’ score for the examination. Academic staff participating in the standard-setting were blinded as to whether questions formed part of the official university written examination or comprised part of this research study.

### Delivery of the MAC examination

#### Participants

Undergraduate students were recruited from two universities: RCSI (Dublin) and QUB (Belfast). In June 2016 all of the RCSI students from the penultimate year of university (during which they complete their paediatric teaching) were invited to attend for a mock examination, 1 week before sitting their university written examination. This mock examination was the ‘MAC’ examination. The following year, between October 2016 and May 2017, all RCSI students from the following year’s cohort were invited to sit the MAC examination at the end of their 6-week paediatric clinical attachment. Between March 2018 and May 2018, QUB students from the penultimate year of the medical course (the year in which they complete their paediatric teaching) were invited to sit the MAC examination at the end of their 6-week paediatric clinical attachment. Due to a delay in receiving ethical approval only 2 out of 5 of the QUB paediatric attachments could be included.

All SHOs currently enrolled in the Irish Basic Specialist Training (BST) [11] scheme, for paediatrics, were approached to sit the MAC examination during the first paediatric training day of the new academic year in October 2016, at which point they had been working in paediatrics for 3 months. The Basic Specialist Training (BST) scheme is a two-year programme completed in Senior House Officer (SHO) posts. Completion of BST in paediatrics is the first step towards becoming a paediatrician in Ireland.

#### Data collection

Each of the examinations took place under standard examination conditions and was invigilated by the study investigator. Written informed consent was obtained and paper examination sheets were distributed to each participant. These were collected and marked at the end of the examination by the study investigator. MAC examination papers were destroyed once the mark had been transferred to the research database.

#### Data analysis

The results of the MAC examination were analysed, using SPSS version 24.0, to determine if participants had reached a clinician-determined minimum accepted competency. Examination results were reported as the mean with standard deviation and median with interquartile range. The proportion of students achieving the standard-set passing score is described. Normally distributed data were analysed using student t-tests with a *p*-value < 0.05 representing statistical significance.

Each student’s undergraduate results in the MAC examination was compared with their university final results in paediatrics using Spearman’s Rank correlation, with a co-efficient of 0.0–0.4 indicating a weak correlation and 0.4–0 indicating a moderate correlation. For RCSI undergraduates, MAC examination results were compared with numerical scores from their official university end of year paediatric written examination. For all undergraduate participants (RCSI and QUB), class rank in the MAC was compared with class rank in the final paediatric exams. Institutional ethical approval did not allow for a direct comparison of the individual results of QUB and RCSI students. However, for the purpose of investigating consistency in the performance of students across two different institutions, we calculated a rank correlation between student’s performance in the MAC examination with their performance in official finals examination and compared this between QUB and RCSI students.

For the SHO participants, results were analysed to determine if there was a difference in the performance between the paediatric junior doctors and the undergraduate students.

## Results

The e-mail request for questions was delivered to 238 out of 247 (96%) members of RCPI. A total of 76 questions (5 duplicates) were contributed by 15 consultants. The first reply arrived on 17/08/2015 and the final reply was received on 27/04/2016. The questions on the MAC examination were from a diverse selection of both general and sub-specialty paediatricians. The questions therefore tested a wide range of common and clinically important areas within paediatrics including seizures, lower respiratory tract infections, growth and emergencies.

Using a modified Angoff technique, 9 members of the RCSI faculty calculated a passing score of 41.2%, equating to a passing score of 13/30 on the MAC examination.

A total of 478 participants were recruited into the study. Of 611 eligible RCSI undergraduates, 366 were recruited into the study over a two-year period (Year 1198/297 [67%] and year 2168/314 [54%]. Of 90 eligible QUB students, 54 (60%) joined the study and 58 out of 62 BST SHOs attending the first study day (93.5%) were enrolled.

Reasons for declining the invitation to sit the MAC examination were not recorded as this was a voluntary extra assessment and not part of the mandatory curriculum.

**Fig. 1:**
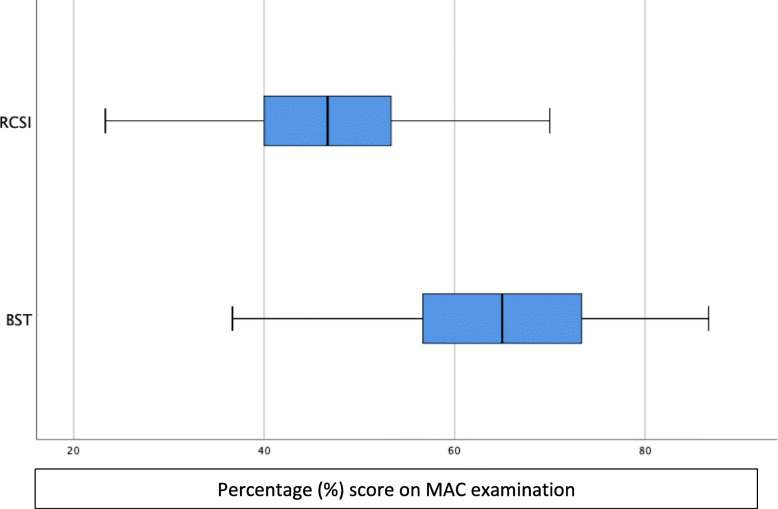
Boxplot of RCSI students and BST SHO scores in the MAC examination

There was no statistical difference in the mean MAC score between year 1 RCSI and year 2 RCSI [*p* = 0.305] (Table 1). Pass rates for official university exams did not differ between groups (year 1 RCSI 96%, year 2 RCSI 97%) but differed significantly from MAC paper scores [year 1 65.2%, year 2 67.9%] (Table 1). The difference between undergraduate RCSI and BST SHO scores was significant [*p* < 0.01] (Table 1). For the RCSI student group the median score was 46.7% (IQR 13.3) and for the BST group the median score was 65% (IQR 18.7) (Fig. 1). No candidate achieved full marks.

**Table 1:** Summary of MAC examination scores

	RCSI year 1 (*n* = 198)	RCSI year 2 (*n* = 168)	RCSI combined (*n* = 366)	BST SHOs (*n* = 58)
Mean score % (SD)	46.3 (10.2)	45.4 (9.6)	45.9 (9.7)	64.2 (11.8)
Proportion ‘passed’ %	65.2	67.9	65.4	n/a

A Spearman’s rank correlation co-efficient showed a moderate but statistically significant positive correlation between students results in their official university examinations and their score in the MAC examination for RCSI students but only a weak correlation for QUB students (Table 2.)

**Table 2:** Correlation of class rank achieved in MAC examination and the same candidate’s class rank achieved in their official university paediatric examination (Spearman’s rank correlation)

	Rank correlation co-efficient	*P* value
Year 1 RCSI (*n =* 198)	0.55	< 0.01
Year 2 RCSI (*n =* 168)	0.39	< 0.01
Combined Year 1 and Year 2 RCSI (*n =* 366)	0.41	< 0.01
QUB (*n* = 54)	0.30	0.029

## Discussion

The literature includes examples of ‘minimal essential requirement’ MCQ examinations designed to assess competence [12]. However, there does not appear to be a comparable study using MCQ items solely from ‘non-faculty’ clinicians and, in this regard, this study introduces a novel concept.

While the content of examination questions is important, the priority is to ensure that medical colleges produce high quality physicians. A paper by Christensen et al. in 2007 highlights the importance of an ‘outcome-based’ approach in medical education, compared to a process/content orientation. However, there are some reservations, as worry is expressed about the taxonomy of learning in pure outcome-based medical education, in which student assessment can be a major determinant for the learning process, leaving the control of the medical curriculum to medical examiners [13]. In the development of the MAC examination, we have designed an examination which is outcome-based but is designed by a broad range of ‘on the ground’ clinicians rather than just academic faculty members.

Another study reported that when undergraduate students wrote the MCQ items (‘peer-written’ questions), the examination results correlated well with their official pediatrics examination but were overall of a tougher standard [14]. A recent study of surgical students showed that the scores from a peer-written examination correlated well with other independent measures of knowledge such as United States Medical Licensing Examination (USMLE) and National Medical Board Examination (NMBE) examinations and also with the universities’ surgery clerkship examination [15]. This is comparable with results from the MAC examination which have been shown to correlate well with students’ marks in the official RCSI final paediatric examinations.

Potts et al. on designed a summative assessment based on six core paediatric objectives [16]. Passing all items was a requirement and failure required remedial oral examination of any failed items. When ‘pre-warned’ of the emphasis on these aspects of the curriculum, student performance in this examination significantly improved compared with the previous year (control group). However, this same cohort of students performed worse on the NBME paediatric subject examination. In the Potts study, poor student performance in the NBME was likely due to their attention being drawn towards passing the new ‘in-house’ summative examination, the consequence of which was missing many key components of the curriculum set out by the NBME. The study did not answer whether or not these students were poorly prepared to be paediatricians, but simply highlights the distinct difference in the two curricula. This raises a few important points; assessment drives student learning and therefore needs to be reflected in the curriculum. Also, students are capable of meeting an agreed learning objective when specifically prepared for it. However, if the curriculum they are taught is not reflected in all of their summative testing it may prove detrimental. This is comparable with our study in that the students performed poorly in the MAC compared with the official RCSI examination, the curriculum for which they were familiar with and were specifically prepared for. When set a different test, albeit on the same subject, the percentage scores were significantly lower.

### Standard setting MAC examination

The result of the standard-setting process was that the MAC examination was given a ‘passing score’ of 41.2% (13/30). This is relatively low for a ‘finals’ high-stakes examination especially considering that the intention was to design an examination with questions which were deemed ‘must know’, ‘basic knowledge.’

Although in many institutions cut scores are often between 50 and 70% [17], there is an argument that cut scores should be higher. The higher the cut score, the smaller the chance of false positives (i.e. candidates able to pass the examination by guessing the answers). This is of particular importance when the licensure will be in a task, failing which will cause serious effect on the individual or society using the service, such as in final medical examinations [18].

### Interpretation of results

We must consider why the students found the MAC questions so difficult and why so many did not achieve the passing score.

It is unlikely that the standard-set for the exam was too high, as the passing score is already below usual thresholds. It is possible that the students’ level of paediatric knowledge reflects the RCSI curriculum, indicated by the high passing rates (96–97%) of the same students in the RCSI examination. Their relatively poor results in the MAC examination have therefore highlighted a significant gap between the RCSI curriculum and the knowledge required for the MAC examination (i.e. what the non-faculty clinicians expect them to know). The poor results in the MAC examination do not indicate that these students will make poor paediatric doctors, but it highlights a potential difference between the RCSI curriculum, and the ‘hidden’ curriculum as determined by non-academic clinicians.

Reassuringly, the paediatric SHOs about to embark on their paediatric career performed significantly better than the medical students. This is an important finding as the MAC examination was designed as a test of knowledge required for ‘on the ground’ clinical practice. In Ireland, paediatric training can commence at postgraduate year 2 (graduates complete a 1 year ‘internship’ in general medicine/surgery during which time they apply for subspecialty training to commence the following year). The majority, but not all, of the participating SHOs would therefore have had 2 more years of clinical experience (1 in their final year of undergraduate study and a 1-year internship). These participants appeared to have benefitted from the extra clinical experience. However, their results still did not match the “must know” standard initially expected by the clinical paediatricians setting the questions.

#### Why was the MAC examination result standard set so low if the questions were meant to be ‘must know’ ‘basic’ knowledge?

This reflects a difference in opinion of the expected standards between faculty members and non-academic clinicians, with the latter seemingly expecting a higher level of knowledge. However, perhaps rather than a ‘higher level’ of expected knowledge, non-academic clinicians expected a different type of knowledge. It is possible that an undergraduate focus on traditional ‘textbook’ facts did not align with the clinicians’ focus on practical aspects of the job, which are particularly relevant to everyday clinical practice. This potential difference in knowledge or focus warrants further investigation at undergraduate level and possibly intervention at early postgraduate level for those planning to practice in paediatrics. There is a move in some third-level institutions to revisit the structure of their undergraduate teaching to increase focus on clinical practice and the broader non-clinical skills required by the physicians [19].

All Irish medical schools have recently collaborated to develop a national undergraduate paediatric curriculum. This will go some way to standardising the knowledge acquired by Irish graduates and is an opportunity to revisit how undergraduate programs are taught. This process should incorporate the views of a wide range of ‘non-academic’ paediatric clinicians to ensure that it can bridge the gap between what is taught and assessed at undergraduate level and what is practically important in the workplace. This study highlights the difficulty in attempting to deliver an undergraduate course that both establishes a core of basic paediatric knowledge and prepares a student for the postgraduate clinical environment. However undergraduate medical education is not merely about the transfer of knowledge to future medical practitioners. It is also about developing transferrable general clinical and non-clinical skills required for good medical practice, including Human Factors, and engendering the skills for lifelong self-directed learning. It may be that bridging this ‘gap’ is not necessarily the responsibility of the university that is preparing graduates to work as general physicians rather than subspecialists, but rather the postgraduate training bodies should possibly be identifying ways in which this type of knowledge is provided and assessed prior to entering the training scheme. This could be delivered in a short induction course and the transitional period of assistantship that many universities now have in place would seem a suitable time to do this. It is anticipated that the results of this study can inform the content of transitional interventions to better prepare them for practice.

#### Did the students perform differently from year to year?

There was no significant difference between the results obtained in the MAC examination between either year of RCSI students, despite the fact that 1 year had the assessment at the end of their paediatric rotation and the other at the end of the academic year. In addition, the fact that two large groups of students obtained such similar results in the exam suggests that this examination is reproducible from year to year.

#### Did students perform differently in their official RCSI end of year examinations compared with how they performed in the MAC examination?

A statistically significant positive correlation between an individual’s MAC score and their score from official RCSI paediatric final assessments demonstrates convergent validity to this new type of assessment.

### Quality of university examinations

Concerns have been raised that the quality of university examinations may not always be sufficient for high-stakes decision-making in clinical practice [20]. Studies have shown that undergraduate medical examinations can be of relatively low quality and that the quality of written examination questions can be significantly improved by providing question writers with formal training [21, 22]. It may be an unrealistic target to expect a large group of ‘non-faculty’ clinicians to undertake extra training in examination writing. A potential solution to this problem would be to encourage ‘non-faculty’ clinicians to provide the question content, in any format they feel most comfortable with, and then to deploy a team of trained academics to revise these questions into a more suitable format and improve their psychometric properties. In fact, this is how the Royal College of Pediatrics and Child Health (RCPCH) generate their examination questions. They set up question-setting groups throughout the country, headed by a member of faculty but attended by non-faculty consultant paediatricians and senior registrars. These questions are then reviewed by the theory examiner team at ‘board meetings’, at which point the questions are revised to be included in a potential bank of ‘live’ questions for use in written examinations.

### Study limitations

There were 15 consultant clinicians providing 71 questions for the MAC examination. It is possible that there would have been even greater breadth and diversity to the questions if there had been a greater number of paediatricians contributing questions. The results of this study may have been influenced by the fact that it relied upon volunteers to provide questions. Therefore, these consultants have self-selected to a certain degree, and our sample may not accurately reflect the opinion of the ‘average’ paediatric clinician. However, their contribution is extremely valuable, as these individuals were sufficiently motivated to contribute to this work.

The official RCSI written examination has 150 test items and therefore the MAC examination, with only 30, is testing a smaller sample of knowledge. We appreciate that this has limited our results. However, the questions used covered a range of topics within paediatrics and represent a finite amount of ‘basic, must know knowledge.’

Both the undergraduate students and SHOs sat the exam voluntarily, and so the results may reflect a more motivated population than the overall cohort. In the SHO cohort, the 93% response rate makes it unlikely that this would have an important effect. In the undergraduate cohort, the proportion of possible candidates volunteering for the exam was lower, so the chances of selection bias are greater. However, there was a significant positive correlation between their MAC results and their official university results. As these rankings did not merely cluster at the top of the class, it is clear that it was not just the highest achieving students who had volunteered to do the exam.

## Conclusion

This study suggests there is a knowledge disparity between what is taught and assessed in the undergraduate domain and what is expected as essential knowledge in the postgraduate domain. Increasing co-operation between academic and experienced non-academic clinicians should help to bridge this gap. Transition interventions such as assistantships and work shadowing would seem to provide a platform for this. It is anticipated that studies such as this will help inform the content of interventions to ensure that future junior paediatric doctors are optimally prepared for practice.

## Data Availability

The datasets during and/or analysed during the current study available from the corresponding author on reasonable request.

## Abbreviations

GMC: General Medical Council
UK: United Kingdom
SHO: Senior House Officer
BST: Basic Specialist Training
RCPI: Royal College of Physicians in Ireland
RCSI: Royal College of Surgeons in Ireland
QUB: Queens University Belfast
MAC: Minimum Accepted Competency
MCQ: Multiple Choice Question
USMLE: United States Medical Licensing Examination
NBME: National Board Medical Examination
